# Extracellular Vesicle Activation of Latent HIV-1 Is Driven by EV-Associated c-Src and Cellular SRC-1 via the PI3K/AKT/mTOR Pathway

**DOI:** 10.3390/v12060665

**Published:** 2020-06-19

**Authors:** Robert A. Barclay, Gifty A. Mensah, Maria Cowen, Catherine DeMarino, Yuriy Kim, Daniel O. Pinto, James Erickson, Fatah Kashanchi

**Affiliations:** Laboratory of Molecular Virology, George Mason University, Manassas, VA 20110, USA; rbarclay@gmu.edu (R.A.B.); gmensah2@gmu.edu (G.A.M.); mcowen4@gmu.edu (M.C.); cdemarin@gmu.edu (C.D.); ykim78@gmu.edu (Y.K.); dpinto1@gmu.edu (D.O.P.); jericks@gmu.edu (J.E.)

**Keywords:** HIV-1, extracellular vesicle, c-Src, PI3/AKT/mTOR pathway, SRC-1

## Abstract

HIV-1 is a global health crisis that has infected more than 37 million people. Latent reservoirs throughout the body are a major hurdle when it comes to eradicating the virus. In our previous study, we found that exosomes, a type of extracellular vesicle (EV), from uninfected cells activate the transcription of HIV-1 in latent infected cells, regardless of combination antiretroviral therapy (cART). In this study, we investigated the specific mechanism behind the EV activation of latent HIV-1. We found that phosphorylated c-Src is present in EVs of various cell lines and has the ability to activate downstream proteins such as EGFR, initiating a signal cascade. EGFR is then able to activate the PI3K/AKT/mTOR pathway, resulting in the activation of STAT3 and SRC-1, culminating in the reversal of HIV-1 latency. This was verified by examining levels of HIV-1 TAR, genomic RNA and HIV-1 Gag p24 protein in cell lines and primary cells. We found that EVs containing c-Src rescued HIV-1 despite the presence of inhibitors, validating the importance of EV-associated c-Src in latent HIV-1 activation. Lastly, we discovered an increased recruitment of p300 and NF-κB in the nucleus of EV-treated infected cells. Collectively, our data suggest that EV-associated c-Src is able to activate latent HIV-1 via the PI3K/AKT/mTOR pathway and SRC-1/p300-driven chromatin remodeling. These findings could aid in designing new strategies to prevent the reactivation of latent HIV-1 in patients under cART.

## 1. Introduction

Human immunodeficiency virus type 1 (HIV-1) is a retrovirus that has infected more than 37 million people worldwide [1]. There is no cure for HIV-1; however, the introduction of combined antiretroviral therapy (cART) suppresses the replication of the virus and prevents the onset of AIDS. cART lowers HIV-1 viral loads to undetectable levels in plasma but is unable to cure or eliminate latent infected cells [2]. As such, the viral infection reactivates upon the discontinuation of cART, leading to viral rebound and an increase in opportunistic infections and the advancement of HIV-1 into AIDS [3,4]. Latent reservoirs throughout the body, primarily in resting memory CD4^+^ T-cells and macrophages, have proven to be a major hurdle when it comes to completely eradicating HIV-1. Latency is established upon the integration of the viral genome into the host chromosome and is characterized by the inability to detect infectious virions (<50 copies/mL) in plasma [2,5]. The immune system is incapable of distinguishing these latent cells from healthy cells and therefore is unable to target them for elimination. The issue of latent reservoirs has led to the development of the “shock and kill” method, in which latency reversing agents (LRAs) reactivate latent infected cells in order for the immune system and/or cART to eliminate the virus [3,6]. Recently, we reported a biological LRA, exosome from uninfected cells, that activates the transcription of latent HIV-1 [7].

Exosomes are small extracellular vesicles (EVs) (30–120 nm diameter) originating from multivesicular bodies and released from most cell types [8,9]. They were originally thought to only remove cellular waste [10]. However, it has now been shown that exosomes participate in many other essential cellular processes including cell-to-cell communication, immune response, and neuronal functions [9,10,11]. Exosomes contain proteins, miRNAs, RNAs, and lipids, and can transfer these distinctive molecules to neighboring cells, eliciting functional changes in these recipient cells [12,13]. Furthermore, exosomes have been implicated in the pathogenesis of several viruses, such as HIV-1, Ebola virus, Rift Valley fever virus, and Hepatitis C Virus [14,15,16,17]. The content of exosomes differs based on the infection or disease, which makes exosomes potential biomarkers for diseases and infections, including HIV-1, Alzheimer’s disease, Huntington’s disease, and cancer [18,19]. In the case of HIV-1, exosomes from infected cells have been shown to contain HIV-1 viral TAR RNA and viral proteins such as Nef [20,21].

Previously, our lab found that exosomes produced from HIV-1-infected cells deliver HIV-1 products, such as TAR RNA, to neighboring cells, increasing their susceptibility to HIV-1 infection in recipient cells by downregulating apoptosis [20]. Furthermore, TAR can activate NF-κB, a transcription factor associated with HIV-1 activation from latency, by binding to and activating the Toll-like receptor 3 (TLR3) signaling cascade [15]. Interestingly, exosomes from uninfected T-cells and monocytes increased basal HIV-1 transcription in latent infected T cells and monocytes, despite the presence of cART, and corresponded with increased HIV-1 p24 protein levels [7]. Furthermore, we observed increased production of a novel RNA transcript, TAR-*gag*, which we later determined to play a role in regulating viral transcription in infected cells [7,22]. In an effort to better understand how exosomes and EVs could be responsible for activation of latent HIV-1-infected cells, we examined the kinase, cellular Src (c-Src), which was previously reported to be in exosomes [23].

c-Src is a non-receptor tyrosine kinase and a member of the Src family of kinases [24]. c-Src is expressed in many cell types, found in the cytoplasm at the plasma membrane, and involved in signal pathways that result in cellular differentiation, proliferation, survival, angiogenesis, and motility [24,25]. c-Src functions by interacting with other cellular proteins such as cell surface receptors, integrins, Stats, and cell-cell adhesion molecules, culminating in the phosphorylation and activation of downstream targets [26,27]. c-Src has been linked to a number of major pathways, including the RAF/MEK/ERK and PI3K/AKT/mTOR pathways, both of which play a critical role in cell proliferation and survival [26,28,29].

The PI3K/AKT/mTOR pathway, together with c-Src, have been linked to HIV-1 [30]. Activation of Src tyrosine kinases has been observed in HIV-1-infected CD4^+^ T cells within minutes after infection [31]. In addition, McCarthy and colleagues demonstrated that inhibiting c-Src restricts HIV-1 early entry in activated primary CD4^+^ T cells [31]. Tyrosine phosphorylation signaling has been shown to be instrumental for viral entry, actin remodeling, and translocation of the viral preintegration complex (PIC) into the nucleus [32,33,34]. The PI3K/AKT/mTOR pathway is a survival pathway that is activated in response to a number of cellular signals, including the activation of growth factor receptors, amplification or mutation of AKT-1 and PI3K, and exposure to carcinogens [29]. PI3K (phosphatidylinositol 3-kinase) is a downstream effector of receptor tyrosine kinases (RTKs) and regulates several cellular processes including proliferation, growth, apoptosis, and cytoskeletal rearrangement [29]. AKT-1 (protein kinase B) is a serine/threonine kinase which regulates a number of signaling pathways that result in changes in cell proliferation, metabolism, apoptosis, and cell cycle [35]. mTOR (mammalian target of rapamycin) is a serine/threonine kinase that is part of the mTOR complex which integrates and regulates several signals that play a critical role in cellular growth, autophagy, proliferation, transcription, and protein translation [29]. The activation of the PI3K/AKT/mTOR pathway initiates at the cell membrane by the activation of PI3K, tyrosine kinase growth factor receptors such as epidermal growth factor receptor (EGFR), insulin-like growth factor-1 receptor (IGF-1R), cell adhesion molecules such as integrins, G-protein-coupled receptors (GPCRs), and oncoproteins such as Ras [29,36]. This leads to the activation of AKT-1 which directly phosphorylates and activates its main downstream target, mTOR [29]. The PI3K/AKT/mTOR pathway is activated in many forms of cancer and has recently been shown to be manipulated by viral proteins, such as Nef, in order to increase pathogenesis [37].

The aim of this study was to investigate the mechanism behind EV activation of latent HIV-1. Here, we propose that the uninfected EV activation of latent HIV-1 initially starts with EV-associated c-Src being delivered into a recipient infected cell, where it is able to activate the PI3K/AKT/mTOR pathway, eventually leading to the activation and translocation of SRC-1 to the nucleus, promoting a pro-transcription state, which would then allow for increased Cdk9 and RNA polymerase II (Pol II) loading onto the HIV-1 promoter. Our data show that EVs from uninfected T cells contain a catalytically active version of the protein kinase c-Src, which is able to activate EGFR, initiating the activation of the PI3K/AKT/mTOR pathway. Upon inhibiting each of the proteins involved in the PI3K/AKT/mTOR pathway, we found a decrease in HIV-1 transcription, as well as in levels of HIV-1 Gag p24 (associated with mature virions) in both cell lines and primary latent cells. Lastly, we found an increase in the recruitment of p300 and NF-κB in the nucleus of EV-treated infected cells, supporting the increased basal transcription of the HIV-1 promoter.

## 2. Materials and Methods

### 2.1. Cells and Reagents

CEM (uninfected T cells), Jurkat (uninfected T cells), U937 (uninfected promonocytic cell), ACH2 (HIV-1-infected T cells), and U1 cells (HIV-1-infected monocytes) were cultured in RPMI 1640 (Quality Biological; Gaithersburg, MD, USA) complete medium containing 1% L-glutamine, 1% streptomycin/penicillin, and 10% exosome-free fetal bovine serum (FBS) at 37 °C and 5% CO_2_. Exosome-free FBS was obtained through ultracentrifugation at 100,000× *g* for 90 min to remove bovine exosomes. Dasatinib (Sellekchem, S1021; Thermo Fisher Scientific, Pittsburg, PA, USA), Gefitinib (Sellekchem, S1025), LY294002 (Sellekchem, S1105), MK2206 2HCl (Sellekchem, S1078), Rapamycin (Sellekchem, S1039), WP1066 (Sellekchem, S2796), and Bufalin (Cayman Chemicals, 465-21-4; Ann Arbor, MI, USA) were used to treat cells in various experiments. α-c-Src (Santa Cruz Biotechnology, sc-19; Dallas, TX, USA), α-c-Src (p-Y416) (Cell Applications Inc., CC1034; San Diego, CA, USA), α-CD63 (Systems Bioscience, EXOAB-CD63A-1; Palo Alto, CA, USA), α-Hck (Santa Cruz Biotechnology, sc-374100), α-Lck (Santa Cruz Biotechnology, sc-433), α-Fyn (Santa Cruz Biotechnology, sc-434), α-p24 (NIH AIDS Reagent Program, 4121), and α-Actin (Abcam, ab49700; Cambridge, MA, USA) were used in Western blots. α-Pol II (Santa Cruz Biotechnology, sc-899), α-p300 (Santa Cruz Biotechnology, sc-585), α-p65 (Abcam, ab7970), and α-IgG (Santa Cruz Biotechnology, sc-66931) were used in chromatin immunoprecipitation (ChIP) assays.

### 2.2. Infection and Treatment of PBMCs

Three PBMC samples were plated and activated with PHA/IL-2 every other day for a total of one week [7,38]. Prior to infection, EVs were isolated from each PBMC via ultracentrifugation. Cells were then infected with HIV-1 89.6, a dual-tropic strain, with a MOI of 10 and incubated for 72 h. On Day 2 post infection, cells were treated with PHA/IL-2. Following Day 3 post infection, cells were treated with IL-7 and a cART cocktail (equal parts of lamivudine (NRTI), tenofovir disoproxil fumarate (NtRTI), emtricitabine (NRTI), and indinavir (protease inhibitor) at 10 µM each). The cART/IL-7 treatment was repeated every other day for the course of one week followed by treatment with 0.5 µM and 2.5 nM of dasatinib and bufalin, respectively, for 2 h. The EVs isolated prior to infection of PBMCs were added back to the respective PBMCs at a ratio of 1:5000 cell per EV and allowed to incubate for 72 h. Cells were harvested for RT-qPCR, and HIV-1 virions were collected from the cell supernatant for Western blot.

### 2.3. EV Isolation and Ultracentrifugation

CEM and HUT102 cells were grown in complete media supplemented with 10% exosome-free FBS, and exosomes were isolated from 500 mL of cell culture grown in a roller bottle over the course of four weeks. Cells were pelleted by centrifugation at 1000× *g* for 10 min, and the cell supernatant was collected. An additional centrifugation at 2000× *g* for 10 min was used to pellet dead cells and cell debris. The supernatant was collected and ultracentrifugation in a Ti70 rotor (Beckman Coulter; Indianapolis, IN, USA) was performed at 2000× *g* for 45 min, 10,000× *g* for 45 min, 100,000× *g* for 90 min, and 167,000× *g* for 16 h to pellet EVs to obtain 2K, 10K, 100K, and 167K EV populations, respectively. For total EVs, a 100,000× *g* spin was performed for 90 min to pellet all EVs. All pellets were then re-suspended in Dulbecco’s phosphate-buffered saline without calcium and magnesium (PBS), consolidated into a single tube per each EV population, and washed with PBS. The resulting pellet was re-suspended in 300 µL of PBS. All centrifugations were performed at 4 °C.

### 2.4. EV Characterization Using ZetaView

Characterization of EVs isolated from CEM cells and PBMCs via ultracentrifugation was done using the ZetaView^®^ Z-NTA (Nanoparticle Tracking Analysis (Particle Metrix, Inning am Ammersee, Germany) and its corresponding software (ZetaView 8.04.02). One hundred nanometer polystyrene nanostandard particles (Applied Microspheres; Leusden, Netherlands) were used to calibrate the instrument prior to sample readings at a sensitivity of 65 and a minimum brightness of 20. For each measurement, the instrument pre-acquisition parameters were set to a temperature of 23 °C, a sensitivity of 85, a frame rate of 30 frames per second (fps), and a shutter speed of 250. CEM and PBMC EVs were diluted in PBS prior to being loaded into the cell. Measurements by ZetaView were taken at 11 different positions throughout the cell, with 3 cycles of readings at each position. The mean, median, mode (indicated as diameter) sizes, and concentration were then calculated by the ZetaView software and analyzed using the same software and Microsoft Excel 2016. Data corresponding to CEM and PBMCs EV characteristics including concentration and size can be found in the supplementary data (Figure S1).

### 2.5. Nanoparticle Capture of EVs/Virions

One milliliter of 5-day-old CEM, Jurkat, U937, THP-1, and HeLa JC53 cell supernatant was harvested and centrifuged at 1200× *g* for 10 min to remove cells. Then, 30 µL of a 30% slurry of NT80/82 particles (EVs; Ceres Nanosciences, Inc.) or a 50% slurry of NT86 (virions; Ceres Nanosciences Inc.; Manassas, VA, USA) were added to the supernatant and rotated at 4 °C overnight. This was followed by another centrifugation at 12,000× *g* for 10 min. The resulting pellet was washed with 1 mL of PBS and re-suspended in 15 µL of Laemmli buffer for Western blot analysis.

### 2.6. Cell Transfection

Four microliters of 100 µM siGENOME SMARTpool siRNA (Horizon Discovery; Cambridge, MA, USA) against c-Src was added to Attractine reagent at a ratio of 1.5 µL Attractine:1 µL of siRNA, mixed, and incubated for 1 h at room temperature. Log phase ACH2 and CEM cells were then placed in 50 µL of fresh, FBS-depleted media within a microcentrifuge tube. siRNA against c-Src was then added to the cells at a final concentration of 100 nM and incubated for 72 h at 37 °C. ACH2 cells were then transferred to a 24-well plate and 3% FBS media was added to the samples whereas siRNA treated CEM cells were subjected to a 100K spin to isolate EVs. An EV titration of either 10^9^ or 50^9^ CEM EVs were added to the ACH2 cells for one experiment while siRNA-treated CEM EVs were added to serum starved (1% FBS) U1 and ACH2 cells for another experiment at a cell to EV ratio of 1:10^3^, 1:10^4^, or 1:10^5^. All samples were allowed to incubate at 37 °C for 48 h. Cell supernatants were collected for NT86 pulldown and Western blotted for HIV-1 Gag p24.

### 2.7. Cell Lysis

Cells were centrifuged at 1000–1200× *g* for 10 min, and the supernatant was removed. The resulting pellet was re-suspended in an appropriate amount of lysis buffer (50 mM Tris-HCl at pH 7.5, 120 mM NaCl, 5 mM EDTA, 0.5% NP-40, 50 mM NaF, 0.2 mM Na_3_VO_4_, and one complete protease cocktail tablet), and vortexed. Cells were incubated on ice for 20 min with vortexing every 5 min. Cell debris was removed by centrifuging at 15,000× *g* for 10 min at 4 °C. Total protein concentration on the resulting lysate was performed by Bradford assay (Bio-Rad; 5000002) using the manufacturer’s instructions.

### 2.8. Western Blot Analysis

Samples were loaded onto a 4–20% Tris-glycine gel (Invitrogen; Pittsburg, PA, USA) and run at 180V. An overnight transfer of proteins onto Immobilon membranes (Millipore; Burlington, MA, USA) at 50mA was then performed. Membranes were then blocked for 30 min with PBS containing 0.1% Tween 20 (PBS-T) and 5% dry milk at 4 °C. Membranes were incubated overnight at 4 °C with the appropriate primary antibody against specified proteins. The next day, the membranes were washed twice with PBS-T and incubated with appropriate HRP-conjugated secondary antibody in PBS-T for 2 h at 4 °C. Membranes were then washed twice with PBS-T and once with PBS. Membranes were developed with Clarity Western ECL Substrate (Bio-Rad; Hercules, CA, USA) and visualized by the Molecular Imager ChemiDoc Touch system (Bio-Rad).

### 2.9. Cytotoxicity Assay

Fifty thousand cells in fresh media were plated in triplicate on a 96-well plate, followed by treatment with inhibitors. Cells were incubated for 48 h and inhibitor treatments were assessed for cytotoxicity using Cell-Titer Glo reagent Luminescence Viability Kit (Promega; Madison, WI, USA) according to the manufacturer’s instructions. RPMI medium alone was used as background in order to normalize values.

### 2.10. RNA Isolation and RT-qPCR

Total cellular RNA was isolated using Trizol reagent (Invitrogen) per the manufacturer’s instructions. A cDNA library was then created using the GoScript reverse transcription system (Promega) following the manufacturer’s instructions with either TAR Reverse (5′-CAA CAG ACG GGC ACA CAC TAC-3′, Tm  =  58 °C) (for HIV-1 TAR RNA) or Envelope Reverse (5′-TGG GAT AAG GGT CTG AAA CG-3′; Tm  =  58 °C) (for HIV-1 genomic RNA) used as a reverse primer. qPCR analysis was then performed using 2 µL of undiluted cDNA per sample with iQ Supermix (Bio-Rad) and the following primers: TAR Reverse and TAR-Forward (5′-GGT CTC TCT GGT TAG ACC AGA TCT G-3′, Tm  =  60 °C). 8E5 (CEM T cell line containing a single copy of HIV-1 LAV provirus per cell) DNA serial dilutions were used as DNA standards. The following PCR conditions were utilized: one cycle at 95 °C for 2  min, 41 cycles at 95 °C for 15  s and 58 °C for 40  s. DNA absolute quantification was determined based on the cycle threshold (Ct) value compared to the standard curve. All PCR reactions were carried out in triplicate using the CFX96 Real Time System (Bio-Rad).

### 2.11. Kinase Assay

CEM, Jurkat, and U937 supernatant were used to collect EVs (NT80/82), lysed, and immunoprecipitated (IP) overnight with antibodies against IgG and c-Src (5µg). Complexes were then precipitated with A+G beads (Calbiochem; San Diego, CA, USA) for 2 h at 4 °C. IPs were then washed twice with TNE buffer (Tris (pH7.5), NaCl, EDTA) and kinase buffer prior to incubation with γ-32P ATP and purified histone H1. Reactions were incubated at 37 °C for 30 min followed by the addition of Laemmli buffer. The samples were separated by reducing SDS-PAGE on a 4–20% Tris–glycine gel. Gels were stained with Coomassie blue, destained, and then dried for 2 h. The gels were exposed to a PhosphorImager cassette and analyzed utilizing Molecular Dynamic’s ImageQuant Software.

### 2.12. ChIP Assay

ChIP assays were performed as defined previously [7]. Briefly, cells were crosslinked and processed using the Imprint Chromatin Immunoprecipitation Kit (Sigma; St Louis, MO, USA). The samples were then sonicated and antibodies were used to immunoprecipitate the resulting mono-disomes by rotating overnight at 4 °C. The resulting complexes were captured using a 50% (*v*/*v*) protein A-Sepharose/protein G-Sepharose mix following a 2-h rotation at 4°C. Samples were then washed, proteins degraded by addition of Proteinase K (800 U/mL), and crosslinking reversed using reversing solution (Sigma) following a 90-min incubation at 654 °C. DNA was purified and real time-qPCR was performed using NF-κB site 1 forward primer (5′-TTC CGC TGG GGA CTT TCC-3′; Tm  =  58  °C) and TAR Reverse.

### 2.13. Statistical Analysis

Standard deviations were calculated for quantitative experiments using Microsoft Excel. P-values were calculated using a two-tailed student’s *t*-test and were considered to be statistically significant when *p*  <  0.05 (*), of greater significance when *p*  <  0.01 (**), and of greatest significance when *p*  <  0.001 (***).

## 3. Results

### 3.1. c-Src Is Present in Multiple Cell Lines and Different EV Populations

**Figure 1:**
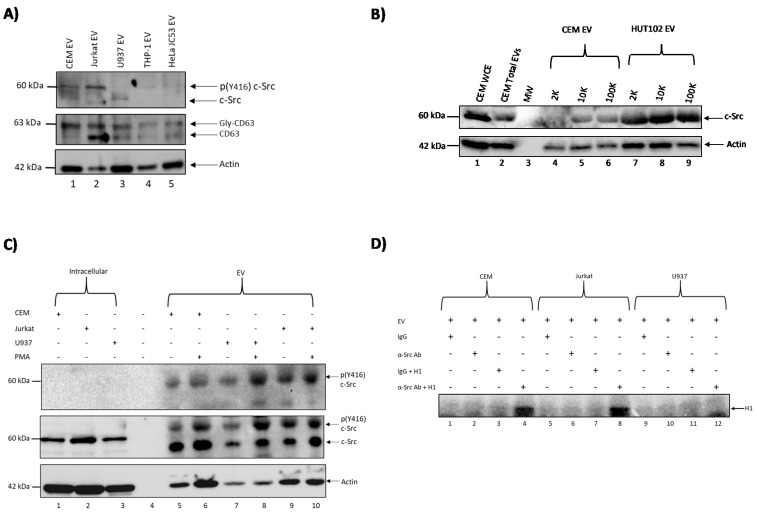
c-Src is present in extracellular vesicles (EVs) in different cell lines and EV populations. (**A**) One milliliter of cell supernatant from CEM, Jurkat, U937, THP-1, and HeLa JC53 cells was collected and treated with Nanotraps (NT80/82) prior to rotating for 72 h at 4 °C to concentrate EVs. EVs were then Western blotted for the presence of c-Src and CD63 (exosomal marker). Actin was used as a loading control. (**B**) 2K, 10K, and 100K EV populations were isolated from CEM and HUT102 cells via ultracentrifugation and Western blotted for c-Src. CEM whole cell extracts (WCEs) and CEM total EVs (comprising of 2K, 10K, and 100K EV populations) were used as positive controls, while actin served as a loading control. (**C**) CEM, Jurkat, and U937 cells were treated with 100 nM phorbol 12-myristate 13-acetate (PMA) and allowed to incubate for five days at 37°C with a second PMA treatment on Day 3. EVs were then concentrated using NT80/82 and rotated overnight at 4°C. Samples were Western blotted for c-Src and actin. WCEs were used as positive controls for Western blots. Actin was used as a loading control. (**D**) CEM, Jurkat, and U937 EVs were collected, lysed, and immunoprecipitated (IP) overnight with antibodies against IgG and c-Src. Complexes were then precipitated with protein A+G agarose beads for 2 h at 4 °C. IPs were washed twice with TNE (Tris (pH7.5), NaCl, EDTA) buffer and kinase buffer prior to incubation with γ-32P ATP. The IPs were then used for in vitro kinase assays using histone H1 as a substrate.

We have previously shown that exosomes from uninfected cells can increase HIV-1 transcription in latent infected cells [7]. This was accompanied by an increase in mostly short transcripts but also some increase in HIV-1 Gag p24 protein levels, indicating production of low-level infectious HIV-1 virions. We also observed that exosomes from uninfected cells caused increased loading of phosphorylated RNA polymerase II (Pol II) (Ser2/Ser5) and Cdk9 on the HIV-1 promoter, which accounted for the increased HIV-1 transcription [7]. We therefore asked whether an EV-associated kinase could be responsible for the aforementioned effect. It has previously been shown that c-Src kinase is involved in HIV-1 transcription [31,39,40]. As such, we postulated that c-Src could contribute to the EV reversal of HIV-1 latency. To test this hypothesis, we captured EVs from several uninfected cell lines, including CEM (T cell), Jurkat (T cell), U937 (monocyte), THP-1 (myeloid), and HeLa JC53 (epithelial cell expressing CD4); using Nanotraps (NT80/82) followed by washes, and looked for the presence of c-Src, CD63, and actin proteins using Western blots. The results of such an experiment are shown in Figure 1A, where multiple c-Src proteins are present in T cell EVs while myeloid-related and HeLa EVs showed mostly one form of c-Src. We postulated that the higher band observed in T cells, THP-1, and HeLa EVs may be modified c-Src (i.e., phosphorylated). CD63 was used as a control for the presence of EVs in these supernatants. Additionally, we tested for the presence of c-Src in various EV populations using differential ultracentrifugation (2K, 10K, and 100K) [41]. The results in Figure 1B demonstrate that c-Src was present in all the tested EV populations especially in the 100K EV population (Figure 1B lanes 6 and 9).

In order to confirm that the upper c-Src protein observed in Figure 1A was phosphorylated c-Src, we treated CEM, Jurkat, and U937 cells with PMA (phorbol 12-myristate 13-acetate), an activator of c-Src [42], on Days 1 and 3 over the course of five days prior to collecting the cell supernatant. EVs from CEM, Jurkat, and U937 cell lines were then concentrated using Nanotraps (NT80/82) and Western blotted for the presence of phosphorylated c-Src (Y416), total c-Src, and actin. An antibody against phosphorylated c-Src (Y416) was used, since when c-Src is phosphorylated at Y416, it is catalytically active and able to phosphorylate downstream proteins such as EGFR [43,44]. The data in Figure 1C show that EVs from T cells (CEM and Jurkat) and monocyte/myeloids (U937) do indeed contain phosphorylated c-Src (Y416). Also, as expected, treatment with PMA increased phosphorylation of c-Src (Y416). Total unphosphorylated c-Src was present in the whole cell extracts (Figure 1C lanes 1–3). Collectively, these data suggest that EVs from uninfected T cells and monocytes/myeloids contain activated c-Src (phosphorylated at Y416), which can potentially initiate downstream signaling cascades by phosphorylating other target proteins.

In order to define the signaling cascade that ensues following the uptake of EV-associated c-Src, we examined proteins that are substrates of intracellular c-Src, including histone H1 [45]. The rationale was to test whether EV-associated c-Src is able to phosphorylate histone H1, a known substrate of intracellular c-Src. We therefore performed an in vitro kinase assay using EVs from CEM, Jurkat, and U937. EVs were trapped with NT80/82, rotated at 4 °C for 2 h followed by a wash with PBS and treatment with TNE50 + 0.1% NP40. The resulting supernatants were immunoprecipitated (IP) with either IgG or α-c-Src antibody as the source of kinase. Complexes were then IPed with protein A+G, washed, and used for the in vitro kinase assay using γ-^32^P-ATP and purified histone H1 as a substrate. The results of such an experiment are shown in Figure 1D, where histone H1 was phosphorylated with c-Src from mostly T cell EVs (Figure 1D lanes 4 and 8). Collectively, these results suggest that EV-associated c-Src has the potential to activate known substrates of intracellular c-Src upon EV uptake by cells.

### 3.2. Elucidating the Activation Pathway of Latent HIV-1 by c-Src

We next investigated the signal cascade of proteins linking c-Src with HIV-1 transcription. As EV-associated c-Src was able to phosphorylate histone H1 in vitro (Figure 1D), we hypothesized that EGFR, a receptor tyrosine kinase and a target of intracellular c-Src [46], was the first downstream protein involved in latent HIV-1 activation. Along these lines, others have shown that EGFR could activate the PI3K/AKT/mTOR pathway, which could lead to activation of STAT3 by mTOR [47,48,49,50]. STAT3 is a transcription factor that can promote cell proliferation and apoptosis [51,52]. It has also been shown that STAT3 recruits SRC-1 (not to be confused with the non-receptor tyrosine kinase, c-Src). SRC-1 is a nuclear cofactor involved in chromatin remodeling and the promotion of transcription through recruitment of p300 and SWI/SNF [53,54].

**Figure 2:**
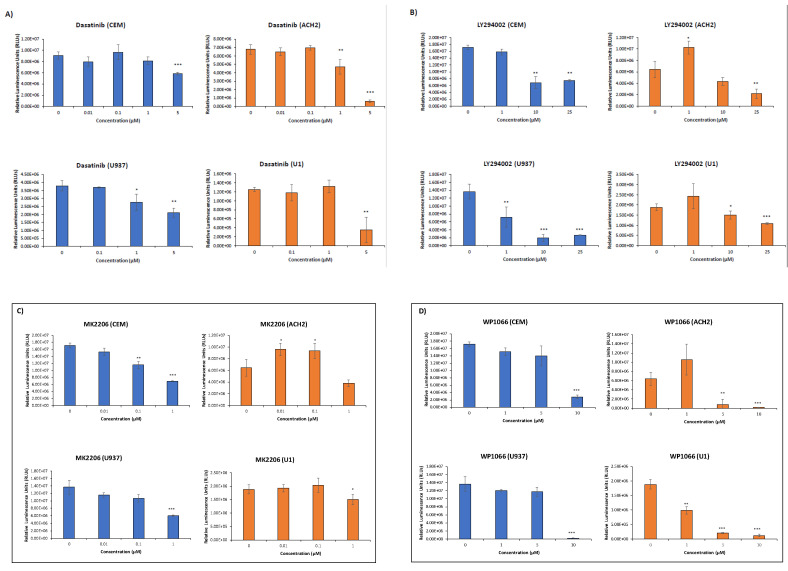

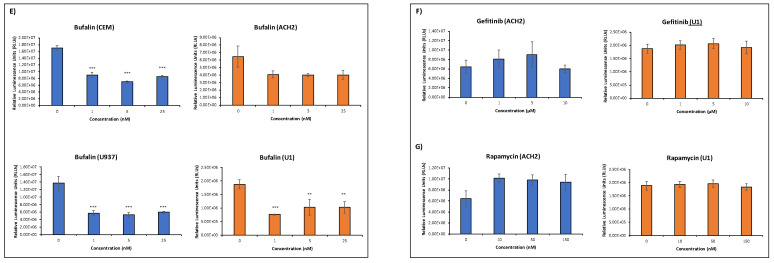
Inhibitor titration of uninfected and latent HIV-1-infected cells. In this process, 5 × 10^4^ cells were plated with different concentrations of kinase inhibitors and allowed to incubate for 48 h prior to a Cell-Titer Glo assay. (**A**) CEM, ACH2, U937, and U1 cells treated with dasatinib (c-Src inhibitor) at 0, 0.1, 1, or 5 µM. (**B**) CEM, ACH2, U937, and U1 cells treated with LY294002 (PI3K inhibitor) at 0, 1, 10, or 25 µM. (**C**) CEM, ACH2, U937, and U1 cells treated with MK2206 (AKT-1 inhibitor) at 0, 0.01, 0.1, or 1 µM. (**D**) CEM, ACH2, U937, and U1 cells treated with WP1066 (STAT3 inhibitor) at 0, 1, 5, or 10 µM. (**E**) CEM, ACH2, U937, and U1 cells treated with bufalin (SRC-1 inhibitor) at 0, 1, 5, or 25 nM. (**F**) ACH2 and U1 cells treated with gefitinib (EGFR inhibitor) at 0, 1, 5, or 10 µM. (**G**) ACH2 and U1 cells treated with rapamycin (mTOR inhibitor) at 0, 10, 50, or 150 nM. Student’s t-tests compared untreated cells with cells treated with drugs. *, *p* < 0.05; **, *p* < 0.01; ***, *p* < 0.001. Error bars, S.D.

In order to test whether each of these proteins (c-Src, EGFR, PI3K, AKT-1, mTOR, STAT3, and SRC-1) were involved in the EV activation of HIV-1 transcription, we treated both ACH2 and U1 cells with inhibitors against each of the aforementioned proteins. Dasatinib, gefitinib, LY294002, MK2206, rapamycin, WP1066, and bufalin were used to target c-Src, EGFR, PI3K, AKT-1, mTOR, STAT3, and SRC-1, respectively [55,56,57,58,59]. To assess the optimal dosage needed for subsequent experiments, we performed a series of drug titrations in both T cells (CEM and ACH2) and monocytes (U937 and U1), and evaluated the viability of the drug-treated cells by Cell-Titer Glo. The results in Figure 2A show that infected cells exhibited less viability when treated with concentrations of dasatinib (c-Src inhibitor) at 5 µM. LY294002 (PI3K inhibitor) resulted in less cell viability in both cell types at 25 µM (Figure 2B). MK2206-treated cells (AKT-1 inhibitor) had less cell viability at a concentration of 1 µM (Figure 2C) while WP1066 (STAT3 inhibitor) showed major loss of cell viability in both cell types at concentrations above 1 µM (Figure 2D). Bufalin caused a loss of cell viability in both cell types starting at 1 nM (Figure 2E). Gefitinib (EGFR inhibitor) and Rapamycin (mTOR inhibitor) showed no significant decrease in cell viability at any of the doses tested in infected cells (Figure 2F,G). Taken together, these results indicate that dasatinib, LY294002, MK2206, WP1066, and bufalin affected infected cells compared to uninfected cells at the dosages tested, suggesting that these inhibitors confer selectivity in infected cells.

### 3.3. EVs Containing c-Src rescue HIV-1 Levels in Inhibitor-Treated Cells

**Figure 3:**
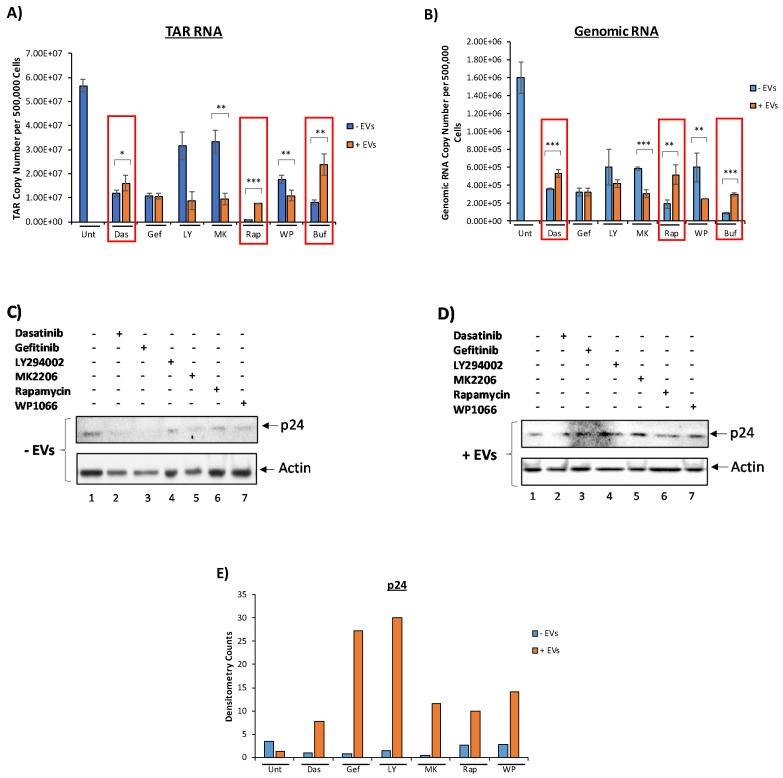
Effect of EVs on the activation of HIV-1 in the presence of various kinase inhibitors. CEM EVs were isolated by ultracentrifugation. Here, 5 × 10^5^ U1 cells were plated and treated with 5 µM dasatinib, 10 µM gefitinib, 10 µM LY294002, 1 µM MK2206, 150 nM rapamycin, or 1 µM WP1066 and allowed to incubate for 48 h. A second drug treatment was then performed. This was followed by a 2-h incubation and a CEM EV treatment. A second EV treatment was performed after a 24-h incubation period. The total ratio of cells to EVs was 1:10,000. Cells were then allowed to incubate for 24 h prior to harvest. Cell supernatant was collected and rotated overnight at 4 °C with NT86. Total RNA was isolated and subjected to RT-qPCR for HIV-1 TAR RNA (**A**) and genomic RNA (**B**). Red boxes indicate increased HIV-1 TAR and genomic RNA levels in EV-treated cells in the presence of dasatinib, rapamycin, and bufalin. (**C**,**D**) NT86-treated samples were Western blotted for HIV-1 Gag p24. U1 WCE was used as a positive control. Actin was used as a loading control. (**E**) Densitometry counts normalized to actin are shown for HIV-1 Gag p24. For all figures, EV untreated samples were used as negative controls. Student’s t-test compared untreated cells with cells treated with drugs. *, *p* < 0.05; **, *p* < 0.01; ***, *p* < 0.001. Error bars, S.D.

To determine whether c-Src, EGFR, PI3K, AKT-1, mTOR, STAT3, and SRC-1 are involved in the signaling cascade leading to the activation of latent HIV-1, U1 cells were plated and treated with inhibitors against each of the aforementioned proteins. We then asked whether EVs containing c-Src could rescue HIV-1 levels despite members of the pathway being inhibited. Our rationale for this experiment was that if EV-associated c-Src was truly involved in the activation of latent HIV-1, the additional c-Src entering the cell should override the inhibition of intracellular c-Src. Briefly, U1 cells were plated and treated with various kinase inhibitors. Cells were allowed to incubate for 48 h followed by a second drug treatment. After a 2-h incubation period, CEM total EVs (comprising of 2K, 10K, and 100K EV populations) were added to cells (1 cell:5000 EVs) and incubated for 24 h. A second EV treatment (1 cell:5000 EVs) was then performed. At the end, the total ratio of cells to EVs was 1:10,000. Cells were then allowed to incubate for 24 h prior to harvest. Cell pellets were prepped for RNA isolation and subsequent RT-qPCR analysis to measure the levels of HIV-1 TAR and genomic RNA while cell supernatants were collected and treated with Nanotraps (NT86) overnight prior to assaying for HIV-1 virions by Gag p24 Western blot. The results from Figure 3A,B show that upon treatment with inhibitors against c-Src, EGFR, PI3K, AKT-1, mTOR, STAT3, and SRC-1, the levels of HIV-1 TAR and genomic RNA decreased in infected cells not treated with EVs. However, upon addition of CEM EVs, cells treated with dasatinib, rapamycin, and bufalin saw increased HIV-1 TAR and genomic RNA levels compared to the non-EV-treated samples (Figure 3A). Similarly, treatment with CEM EVs (containing c-Src) exhibited increased genomic HIV-1 RNA levels in dasatinib, rapamycin, and bufalin-treated cells (Figure 3B). This suggests that EVs containing c-Src were indeed able to rescue HIV-1 TAR RNA levels, despite the presence of high dosages of inhibitors against key upstream proteins implicated in HIV-1 transcription. The mTOR inhibitor, rapamycin, exhibited the most robust decease in HIV-1 TAR RNA levels, while the SRC-1 inhibitor, bufalin, saw the largest decrease in genomic RNA levels. This points to SRC-1 as being more important in the transcription of full-length, coding HIV-1 RNA as opposed to mTOR, which might be more vital in the transcription of shorter, noncoding HIV-1 RNA. Additionally, we assayed for the presence of HIV-1 virions in the cell supernatant. We observed increased levels of Gag p24 in all EV-treated cells despite treatment with inhibitors compared to the non EV-treated counterparts (Figure 3C,D). The increased expression of HIV-1 Gag p24 in EV-treated cells was confirmed by the quantification of the Western blot bands by densitometry analysis normalized to actin (Figure 3E). Collectively, these data indicate that EV-associated c-Src plays a critical role in EV activation of latent HIV-1.

### 3.4. Confirming EV-Associated c-Src Activates Latent HIV-1 in Infected Cells

**Figure 4:**
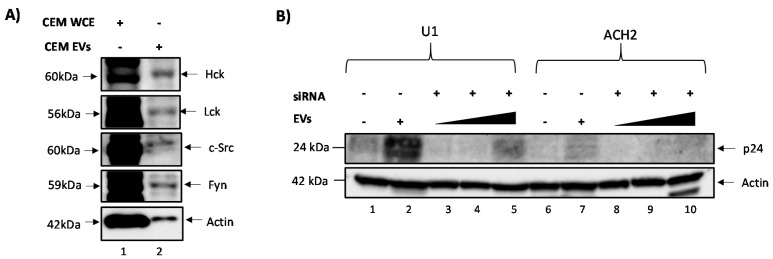

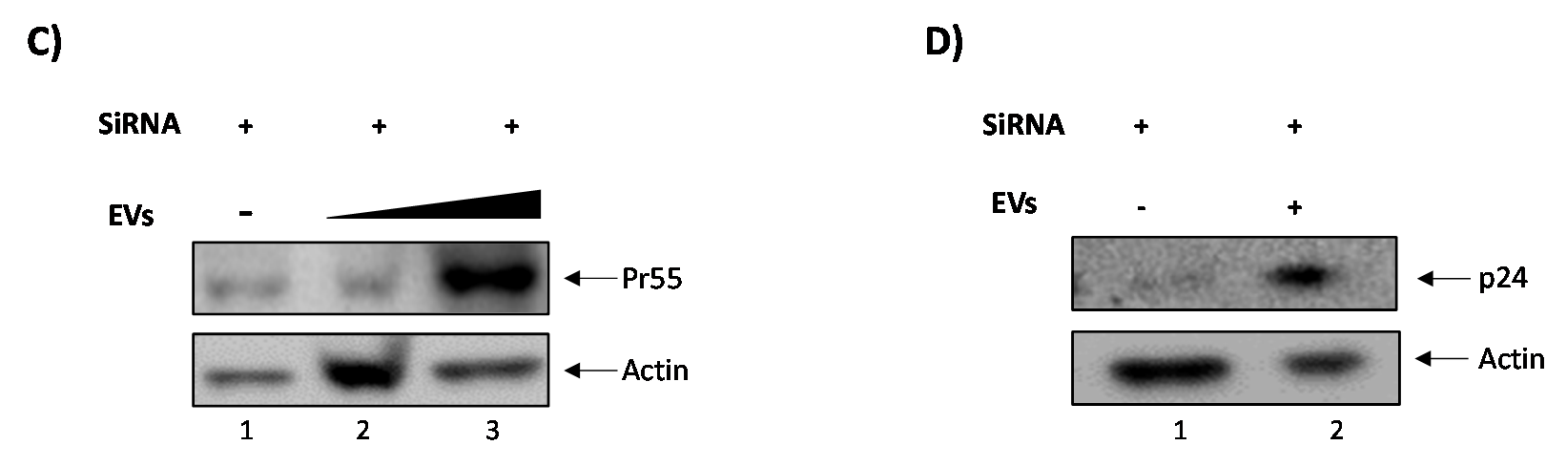
EV-associated c-Src activates HIV-1 in infected cells. (**A**) CEM EVs were isolated via ultracentrifugation (100K) and Western blotted for various src family kinases (lane 2). CEM WCE (lane 1) and actin were used as positive controls. (**B**) Log phase CEM cells were transfected with siRNA (100 nM) against c-Src for 72 h. EVs were isolated using ultracentrifugation (100K), measured, and then added to serum starved (1% FBS) U1 and ACH2 cells at a cell to EV ratio of 1:10^3^, 1:10^4^, or 1:10^5^. Cells were collected 48 h later and Western blotted to assess the levels of newly synthesized p24. (**C**,**D**) ACH2 cells were transcriptional silence by serum starvation. This was followed by intracellular c-Src knockout in ACH2 cells by transfecting cells using siRNA against c-Src. CEM EVs (containing c-Src) were added to ACH2 cells at 0, 10^9^, or 50^9^ EVs (**C**) or 0 or 50^9^ EVs (**D**) and allowed to incubate for 72 h at 37 °C. Samples were then analyzed by Western blot for HIV-1 Gag Pr55 (**C**), Gag p24 (**D**), and actin.

Next, we wanted to confirm that c-Src, rather than other src family member kinases, such as Hck, Lck, or Fyn, was involved in the reactivation of latent HIV-1. The rationale for this experiment was that c-Src might not be the only src kinase packaged into EVs, as other src kinases including Lck has been shown to be present in T cells [60]. This was indeed confirmed by data shown in Figure 4A, where all the src kinases tested (Hck, Lck, c-Src, and Fyn) were present in both CEM whole cell extracts (WCEs) (50 µg) and CEM EVs (10,000 EVs). Another caveat we sought to address was the specificity of dasatinib, which has been shown to inhibit other kinases besides c-Src [61]. As such, we applied an alternate approach of inhibiting c-Src and performed additional experiments using CEM EVs isolated from CEM cells transfected with siRNA against c-Src. Here, latent HIV-1 reactivation was assessed by measuring p24 expression levels in treated and untreated cells using Western blot analysis. The results in Figure 4B show that p24 levels were higher in both U1 and ACH2 cells when c-Src was present in EVs (lanes 2 and 7) compared to the controls (lanes 1 and 6) or when it was treated with a titration of EVs obtained from siRNA-treated CEM cells (lanes 3–5 and lanes 8–10). The reversal of latent HIV-1 was diminished in the absence of c-Src in EVs, suggesting that c-Src kinase may be responsible for the observed reactivation of latent HIV-1 in these infected cells.

Furthermore, we wanted to verify that EV-associated c-Src (not intracellular c-Src) activates latent HIV-1. Our reasoning was that intracellular c-Src, which is present in all cell types [28], could be responsible for the activation of latent HIV-1 and possibly override the effects of EV-associated c-Src, despite the presence of dasatinib. To test whether latent HIV-1 activation was due to EV-associated c-Src (and not intracellular c-Src), we serum-starved ACH2 (infected T cells) cells to force them into transcriptional silence. Intracellular c-Src was then knocked out using siRNA against four different sequences within the c-Src gene. CEM EVs (containing c-Src) were then added to ACH2 cells, which were allowed to incubate for 24 h. Cells were then harvested and Western blotted for HIV-1 Gag Pr55, p24, and actin. The results in Figure 4C reveal increased levels of Gag Pr55 in EV-treated ACH2 cells compared to untreated cells. Furthermore, Pr55 levels were dependent on EV concentration, highlighting the possible correlation between Pr55 expression and the amount of EV-associated c-Src taken up by the cell. The data in Figure 4D show that siRNA knockdown of intracellular c-Src led to minimal Gag p24 expression within infected cells. However, upon EV addition to infected cells, a dramatic increase in p24 expression was observed. Specifically, there was an approximately four-fold increase in the level of p24, indicating the activation of HIV-1. Collectively, these results confirm that EV-associated c-Src contributes to the EV activation of latent HIV-1.

### 3.5. c-Src and SRC-1 Critical in HIV-1 Activation in Primary Cells

**Figure 5:**
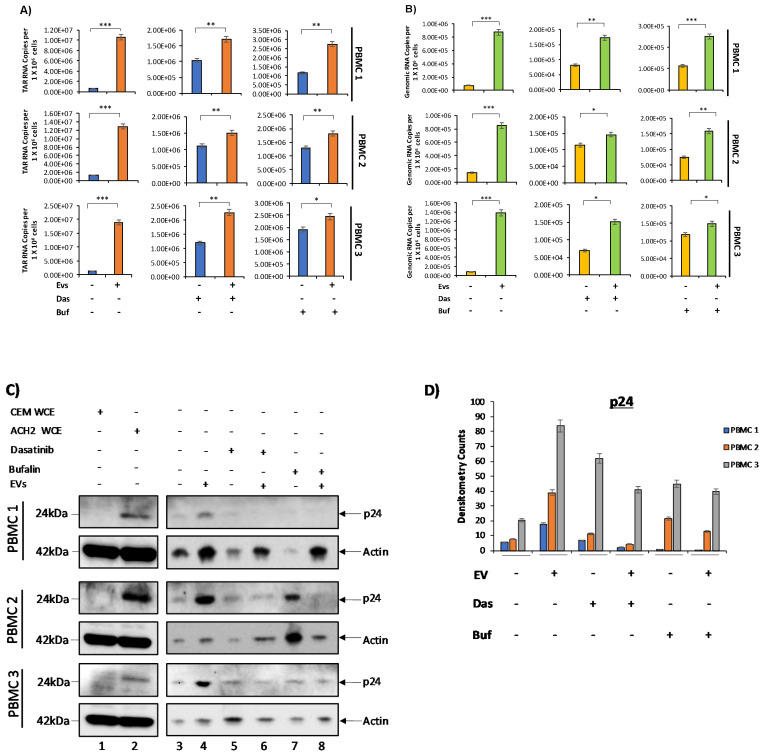
Effects of dasatinib and bufalin on PBMCs treated with cART. Three samples of HIV-1-infected, latent PBMCs were treated with 2.5 nM and 0.5 µM of bufalin and dasatinib, respectively, prior to incubation at 37 °C for 2 h. EVs isolated via ultracentrifugation from each of the PBMCs prior to infection with HIV-1 (89.6) were added back to each of their respective PBMCs. The total ratio of cells to EVs was 1:5000. Cells were then harvested after 72 h of incubation at 37 °C. Total RNA was isolated and subjected to RT-qPCR from cell pellets for HIV-1 TAR (**A**) and genomic RNA (**B**). (**C**) Virions were concentrated from cell supernatant using NT86 and NT80/82 and rotated overnight at 4 °C. Samples were Western blotted for p24. Densitometry counts normalized to actin across all samples are shown for p24 (**D**). CEM and ACH2 WCE were used as a negative and positive control, respectively. Actin was used as a loading control. Student’s t-test compared untreated cells with cells treated with drugs. *, *p* < 0.05; **, *p* < 0.01; ***, *p* < 0.001. Error bars, S.D.

The previous experiments were performed in cell lines, and so we next examined the effect of primary EVs on primary latent infected cells. PBMCs were first grown in PHA/IL-2, and cultured for 8 days, with additional treatments of PHA/IL-2 every other day. On Day 8, EVs were isolated from the cell supernatant using ultracentrifugation (as described in Materials and Methods). We then infected the PBMCs with HIV-1 89.6 dual tropic strain and two days later treated with PHA/IL-2. PBMCs were treated with cART and IL-7 to promote latency and inhibit viral spread. The cART/IL-7 treatment was repeated every other day over the course of one week. Cells were then plated and treated with 0.5 µM and 2.5 nM of dasatinib (inhibitor against c-Src) and bufalin (inhibitor against SRC-1) since they showed the most potent effect on cell lines. Subsequently, the EVs isolated from each PBMC prior to infection were added back to their respective PBMC at a ratio of 1:5000 cell per EV. Cell pellets were assayed for the presence of HIV-1 TAR and genomic RNA via RT-qPCR. Cell supernatants were collected and treated with NT86 and NT80/82 prior to Western blot for the presence of HIV-1 Gag proteins. The results in Figure 5A show a significant increase in HIV-1 TAR RNA levels upon the addition of EVs in all three PBMCs, in agreement with our previous observations [7].

The data in Figure 5B indicate that EVs caused a significant increase in genomic RNA levels in controls, as well as in c-Src and SRC-1 inhibitor-treated cells. This increase was observed across all three PBMCs, suggesting that c-Src in EVs may be sufficient to counteract the effects of the inhibitors and promote viral transcription. We next examined the potential effects of these EVs in translation of viral proteins in the extracellular environment. The data in Figure 5C show an increased level of p24 protein upon the addition of EVs in untreated cells (lanes 3 and 4), which confirms that EVs were able to activate latent virus. In order to further evaluate the expression of p24 in PBMC samples, densitometry analysis of the Western blot bands was performed and normalized to actin (Figure 5D). We did not observe an increase in p24 expression in dasatinib- or bufalin-treated PBMCs upon the addition of EVs. Detection of higher levels of p24 protein in latent infected primary cells may require longer timepoints, beyond 72 h post-activation with EVs. Additionally, in order to determine that the effects observed were not due to cell death, we probed for the presence of caspase-3 and PARP-1 in all three PBMCs via Western blot analysis (Figure S2A,B). The results show the absence of active caspase-3 and PARP-1, indicating a lack of cell death. Taken together, our data indicate that the signaling pathway described above (i.e., c-Src at the early stages and use of SRC-1 at the later stages) may be relevant in human primary cells in addition to cancer cell lines. Therefore, our data may represent how circulating EVs in a patient could promote activation of latent HIV-1.

### 3.6. Increased Basal Transcription Is Driven by NF-κB/p300 Pathway

**Figure 6:**
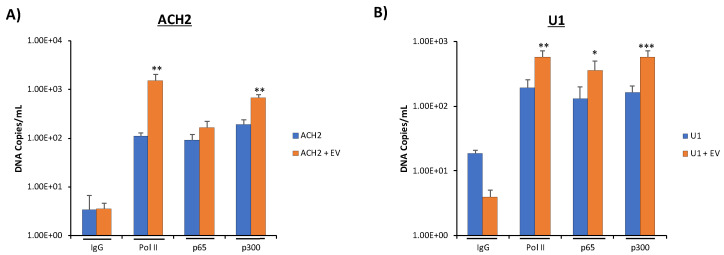
Presence of increased RNA Pol II and p300 on HIV-1 genome. CEM EVs were isolated using ultracentrifugation. ACH2 (**A**) and U1 (**B**) cells were then plated and treated with EVs at 1:5000. Cells were incubated for 24 h and again treated with CEM EVs at 1:5000. Following a second 24-h incubation, cells were harvested and crosslinked prior to ChIP assay using antibodies against IgG, Pol II, p65, and p300. DNA was then quantified using PCR using NF-κB1-2F and TAR +59-R. IgG was used as a background control. A Student’s T-test was used to compare untreated control and EV-treated samples. *, *p* < 0.05; **, *p* < 0.01; ***, *p* < 0.001.

We next examined possible nuclear mechanisms for EV activation of latent HIV-1. To do this, we examined NF-κB, a transcription factor well known to be involved in HIV-1 transcription [62,63]; and p300, a histone acetyltransferase involved in promoting transcription through chromatin remodeling [64,65], loading onto the HIV-1 promoter through the chromatin immunoprecipitation (ChIP) assay. P300 is a downstream target of SRC-1 [66]; therefore, we hypothesized that SRC-1, upon the addition of EVs containing c-Src to HIV-1-infected latent cells, could translocate to the nucleus upon its activation before recruiting p300 to the HIV-1 promoter to drive EV activation of latent HIV-1. To test this hypothesis, we added CEM EVs to both ACH2 and U1 cells and incubated the cells for 48 h. We then harvested the cells, crosslinked, isolated total DNA, used antibodies against NF-κB p65 and p300 for a ChIP assay, and isolated the immunoprecipitated DNA, quantifying it by qPCR. Additionally, we used an antibody against Pol II as a positive control, as we have previously shown that Pol II loading onto the HIV-1 promoter increases with the addition of EVs from uninfected cells [7]. The data from Figure 6A indicate that CEM EV addition to infected ACH2 cells increased p300 recruitment to the HIV-1 promoter by 3.5-fold over the untreated control. As expected, we also observed about a log increase in Pol II loading onto the HIV-1 LTR promoter with the addition of EVs. Interestingly, we observed a slight increase in NF-κB p65 loading onto the HIV-1 promoter upon the addition of EVs to ACH2 cells. This suggests that EV-associated transcription of HIV-1 is driven more by epigenetic-related mechanisms rather than the activation of NF-κB in T cells.

Similar results were obtained in infected U1 cells. Upon the addition of CEM EVs, we observed a 3.4-fold increase in p300 recruitment onto the HIV-1 promoter compared to the untreated control while, as expected, Pol II loading onto the HIV-1 LTR promoter also increased (~3 fold) in the presence of EVs (Figure 6B). NF-κB p65 loading onto the HIV-1 promoter increased in U1 cells by a statistically significant 2.7-fold in the presence of EVs. This indicates that there may be different dynamics in transcription between T cells and monocytes activation of HIV-1 promoter. Collectively, our data suggest that EV activation of latent HIV-1 can be driven by SRC-1 translocation to the nucleus, eliciting p300-related chromatin remodeling.

## 4. Discussion

Extracellular vesicles (EVs) have been shown to play a role in the transport of cargo (i.e., proteins and RNA) between cells, which may elicit functional effects in the recipient cell, such as signal transduction [67,68]. Our past studies have shown a relationship between EVs and viral infection, especially in the context of HIV-1. Specifically, we showed that EVs from infected cells, which contain HIV-1 products (i.e., TAR RNA), can activate TLR3 and suppress innate immune response as well as apoptosis, leading to increased viral pathogenesis or susceptibility in neighboring cells [20,38]. We also showed that EVs from uninfected cells can activate latent HIV-1 by increasing the loading of RNA polymerase II and Cdk9 onto the HIV-1 promoter [7]. Our current data indicate that c-Src, a non-receptor tyrosine kinase involved in a number of cellular processes, can be found in EVs from uninfected cells and that, upon entering into a latent infected recipient cell, could activate the PI3K/AKT/mTOR pathway, which in turn could lead to the activation and translocation of SRC-1 into the nucleus. This would then lead to the recruitment of p300, a chromatin remodeling protein that promotes transcription, thereby resulting in a transcriptionally favorable state of the chromatin and increased loading of RNA Polymerase II onto the HIV-1 promoter, driving latent HIV-1 activation.

We have observed that EVs from various uninfected cell types, including T cells, monocytes, and HeLa cells, contain c-Src (Figure 1A). However, it appeared that T cell EVs in particular contain higher levels of c-Src. Furthermore, this c-Src was observed to be phosphorylated at tyrosine-416 (Figure 1C). This indicates that EV-associated c-Src could be enzymatically active as phosphorylation at tyrosine-416 has been shown to be associated with activated c-Src, which can phosphorylate downstream targets [43,44]. Among these targets are EGFR [46] and histone H1 [45]. Histone H1 was found to be phosphorylated by extracellular c-Src (Figure 1D), leading us to conclude that EGFR could also be a downstream target of EV-associated c-Src, as well as the first kinase to be activated by EV-associated c-Src due to its location at the plasma membrane and its association with the PI3K/AKT-1/mTOR [49]. Furthermore, c-Src was revealed to be present in various EV populations of CEM (Figure 1B lanes 7–9), HUT102 (Figure 1B lanes 7–9), and PC3 cells (Figure S3). Taken together, these data demonstrate that not only is c-Src present in EVs derived from various cell lines, but it is also enzymatically active; and may have a functional effect in recipient cells.

We hypothesized that c-Src, AKT-1, and SRC-1 could be involved in the EV-associated c-Src activation of latent HIV-1. It has been reported that c-Src is linked to AKT-1 and PI3K by EGFR, and AKT-1 is linked to SRC-1 by mTOR and STAT3 [47,48,49,50,53]. Inhibitors against each of the aforementioned proteins were added to infected U1 cells, and we consistently observed that levels of HIV-1 TAR and genomic RNA decreased significantly in the absence of EVs compared to the control (Figure 3A,B). This was accompanied by decreased amounts of HIV-1 Gag p24 found outside the cell (Figure 3C,D). Collectively, this indicates that each of these proteins could be involved in a signal cascade initiated by EV-associated c-Src.

However, when EVs from uninfected cells, previously shown to activate latent HIV-1 [7], were added to infected cells treated with inhibitors against each of the aforementioned proteins, we observed recovery of HIV-1 TAR and genomic RNA levels in cells treated with dasatinib, rapamycin, and bufalin (Figure 3A,B). Furthermore, HIV-1 Gag p24 levels were found to be upregulated when CEM EVs were added to cells, regardless of the type of inhibitor used (Figure 3C,D). In the case of the dasatinib-treated cells, our data show that EV-associated c-Src is able to overwhelm the effect of the inhibitor, thus allowing for HIV-1 to activate/recover. The increased HIV-1 RNA and protein levels in other inhibitor-treated cells in the presence of EVs from uninfected cells imply that there may be other factors within EVs that allow for the activation of latent HIV-1, and that our proposed c-Src/EGFR/PI3K/AKT-1/mTOR/STAT3/SRC-1 pathway is only one of multiple pathways that ultimately lead to EV activation of latent HIV-1. For example, SMARCA1, a SWI/SNF protein involved in promoting transcription, has been shown to be present in EVs and may contribute to chromatin remodeling, leading to the reactivation of HIV-1 from latency [23]. Another protein, S100A9, also associated with EVs, can activate NF-κB and serve as an alternative activator to the PI3K/AKT/mTOR pathway, explaining why we observed increased HIV-1 Gag p24 in the LY294002, MK-2206, and rapamycin-treated cells upon addition of EVs from uninfected cells [69]. Furthermore, Konadu et al. showed the presence of the cytokine IL-4 in EVs; while they showed elevated IL-4 in EVs from HIV-1 infected patients, uninfected patient EVs also contained IL-4, which is known to activate STAT6, which can activate SRC-1 [70,71]. Future studies exploring these possible alternative mechanisms of EV activation of latent HIV-1 should be performed.

Importantly, similar results were observed in three different samples of PBMCs. PBMCs were treated with dasatinib or bufalin. EVs were isolated from each respective PBMC prior to infection. PBMCs with no inhibitor added were used as a negative control. We observed statistically significant decreases of TAR RNA and HIV-1 genomic RNA in all three PBMCs upon addition of dasatinib and bufalin, which was reversed by treatment with EVs. This reversal was significant across all PBMCs. Future experiments will test the use of distinct EV populations, such as EVs of different sizes, cargo, and sources. Additionally, we observed 1-log higher TAR RNA levels than genomic RNA (Figure 5A,B). This is consistent with our previous work which showed the effect of EVs on the transcription of latent HIV-1, where EVs increased the transcription of more short non-coding RNA than long coding RNA [7]. Upon the addition of EVs, we observed statistically significant increases in both TAR RNA and genomic RNA despite the presence of inhibitors (Figure 5A,B), suggesting that the EVs were overriding the effects of inhibitors and rescuing both TAR and genomic RNA levels. When looking at protein translation (HIV-1 Gag p24) in PBMCs compared to cell lines, we noticed a stark difference. Unlike PBMCs, both RNA and protein levels increased in cell lines upon the addition of CEM EVs to inhibitor-treated cells. However, in PBMCs, only RNA levels (TAR and genomic RNA) (Figure 5A,B), and not protein levels (HIV-1 Gag p24) (Figure 5C,D), increased upon treatment with EVs. This suggests that although RNA is being transcribed, it may not be efficiently translated into viral proteins.

It is worth noting that other src kinases besides c-Src could be responsible for activating latent HIV-1 in infected cells, as src kinases such as Lck have been shown to be present in T cells and able to activate downstream targets [60]. Therefore, we assayed and confirmed the presence of additional src kinases, including Hck, Lck, and Fyn, in our CEM EVs (Figure 4A). This led to the important question of whether any of the aforementioned src kinases packaged into EVs could reverse latency in HIV-1 infected cells. Furthermore, the lack of specificity of dasatinib [61] called for a different method of inhibiting c-Src in order to validate our previous data. We therefore utilized EVs derived from CEM cells transfected with siRNA against c-Src to treat recipient cells (U1 and ACH2). The results in Figure 4B confirm that EV-associated c-Src is mostly responsible for the reactivation of latent HIV-1, although other kinases may contribute to increased gene expression at higher EV concentrations. Control EV (without siRNA) resulted in higher reactivation levels (corresponding to p24 expression) in both U1 and ACH2 cells. Along the same lines, the decrease in HIV-1 RNA in the presence of dasatinib observed in Figure 3 could have been caused by intracellular c-Src, rather than EV-associated c-Src. Therefore, we treated latent HIV-1-infected cells with siRNA against c-Src, followed by treatment with EVs from uninfected cells containing c-Src. We observed that upon treatment with siRNA alone, HIV-1 Gag Pr55 and p24 levels were low. However, upon addition of EVs containing c-Src, HIV-1 Gag Pr55 and p24 levels were dramatically increased (Figure 4C,D). Taken together, these data validate our hypothesis that EV-associated c-Src may be responsible for activating latent HIV-1 in infected cells.

Having elucidated a possible mechanism for EV-associated activation of latent HIV-1 through EV-associated c-Src and cell-associated EGFR/PI3K/AKT-1/mTOR/STAT3/SRC-1, we turned our attention to the nucleus to determine a possible mechanism connecting SRC-1 (the cytoplasmic/nuclear link in our hypothesized signal cascade) with the increases in RNA Pol II and Cdk9 loading onto the HIV-1 promoter in response to EV treatment we observed previously [7]. P300, a downstream target of SRC-1, is involved in chromatin remodeling, thereby promoting transcription [64,65,66]. We therefore hypothesized that upon EV addition to cells, p300 binding to the HIV-1 promoter would increase. Subsequent ChIP assays showed increased loading of p300 onto the HIV-1 promoter following treatment of HIV-1-infected latent cells with EVs from uninfected T cells (Figure 6A,B). This was correlated with increased loading of RNA Pol II on the HIV-1 promoter, in agreement with results we published previously [7]. T cells, with the addition of EVs, appear to utilize epigenetic modifications through chromatin remodeling by p300 to drive the reversal of latency, while monocytes appear to utilize both the translocation of cytoplasmic NF-κB p65 to the nucleus and epigenetic modifications related to p300 [2]. This difference in EV activation of latency could be explained by monocytes having different cellular receptors that are activated by EVs compared to T cells. Collectively, however, p300 appears crucial in EV activation of latent HIV-1. This is not surprising as p300 has previously been implicated in HIV-1 activated transcription [72,73]. Based on these data, it may be that EVs could drive full-length HIV-1 transcription in the absence of Tat, especially when paired alongside our previous data, showing increased Cdk9 loading on the HIV-1 promoter in the presence of EVs [7].

Our data could explain several HIV-1-related findings from the clinic. In a 2013 study, 190 patients under cART showed detectable levels (10^1^–10^4^ copies) of viral RNA inside CD4^+^ T cells, despite viral titers being undetectable in plasma [74]. Other studies showed cART-treated patients producing defective and/or mutant viruses over time [75,76,77]. Finally, a survey of brain tissue reservoirs showed the presence of TAR-like sequences in infected patients [78]. These clinical findings may be explained by the EV activation of latent HIV-1, which occurs, at least in part, through the mechanism we have described here. This is because current FDA-approved cART regimens lack a transcriptional inhibitor, focusing only on interfering with HIV-1 at various points in the viral life cycle [79,80]. Therefore, it may be advantageous to explore transcription inhibitors that could supplement cART in better controlling the EV-associated activation of latent HIV-1. One potential drug candidate identified in our study was WP1066, an inhibitor against STAT3 [59]. We identified WP1066 as being a suitable candidate for further testing as we performed a series of cell viability assays in HIV-1-infected cells using inhibitors, at differing concentrations, against each protein candidate in our proposed c-Src/EGFR/PI3K/AKT-1/mTOR/STAT3/SRC-1 signal cascade (Figure 2). Dasatinib, LY294002, MK-2206, WP-1066, and bufalin all showed killing of HIV-1-infected cells at high concentrations. The results showed dasatinib, an FDA-approved drug used to treat chronic myeloid leukemia [28], had selective killing of HIV-1-infected T cells at 1 µM, while WP1066, a drug currently undergoing clinical trial to treat malignant glioma and brain metastasis [81], had selective killing of both infected T cells and monocytes at 5 µM (Figure 2A,D). Based on these data, WP1066 could be a promising candidate to supplement cART, as it was shown to act on both infected T cells and infected monocytes, known latent reservoirs of HIV-1 [2]. Further studies need to be conducted to explore this avenue.

**Figure 7:**
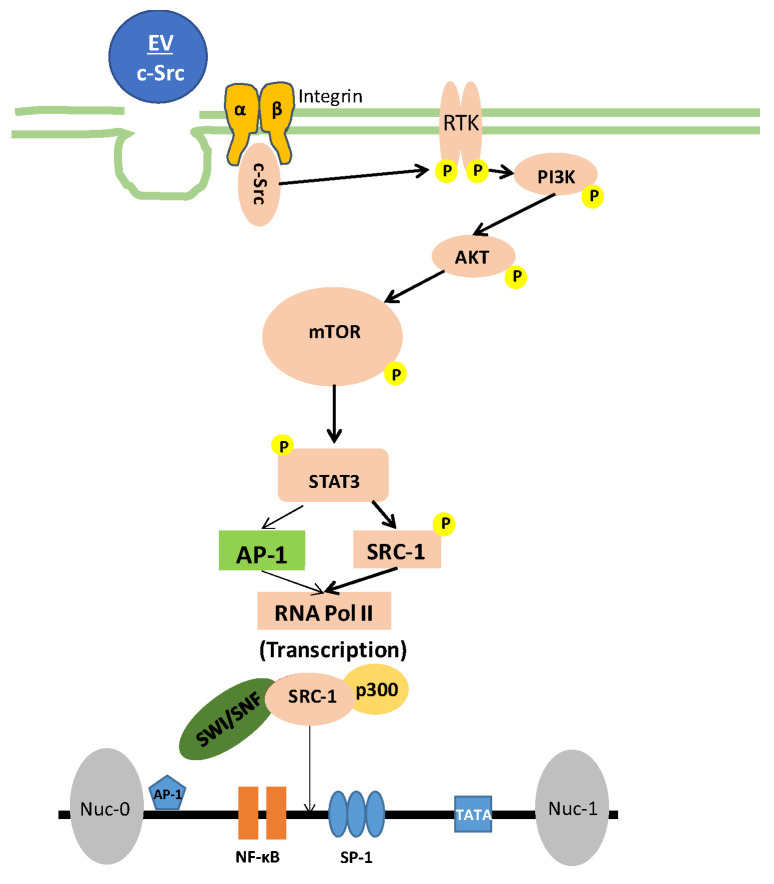
Proposed model for EV activation of latent HIV-1. Uninfected EVs from healthy cells are taken up by HIV-1 latent infected cells, leading to the phosphorylation and activation of receptor tyrosine kinases (RTK) by c-Src. RTK then activates PI3K, which in turn activates AKT. Following activation, AKT phosphorylates mTOR, resulting in the phosphorylation and activation of the transcription inducer, STAT3. STAT3 then recruits the cofactor SRC-1, before translocating to the nucleus and promoting HIV-1 transcription by recruiting p300 (promotes chromatin remodeling). NF-κB and RNA Pol II increase loading onto the HIV-1 promoter, resulting in the transcription of latent HIV-1.

Overall, we have shown that EVs from uninfected cells contain activated c-Src (phosphorylated at tyrosine-416) that can, in cells, activate a signaling cascade involving EGFR, the PI3K/AKT-1/mTOR pathway, STAT3, and SRC-1. SRC-1 can then translocate to the nucleus, recruiting p300 to the HIV-1 promoter, which can then drive chromatin remodeling. This would enable a more open chromatin state, promoting increased loading of RNA Pol II onto the HIV-1 promoter and allow for activation of latent HIV-1 (Figure 7). Inhibition of c-Src, EGFR, PI3K, AKT-1, mTOR, STAT3, and/or SRC-1 led to decreased levels of HIV-1 RNA (both short and long transcripts) inside the cell as well as decreased HIV-1 Gag p24 in the cell supernatant, confirming the importance of these proteins in latent HIV-1 activation. Collectively, this leads us to the conclusion that EV-associated c-Src is important in driving EV activation of latent HIV-1. However, c-Src is likely not the only kinase within EVs that can activate HIV-1 as the inhibition of proteins downstream of c-Src (i.e., SRC-1) was still overridden by the addition of EVs to infected cells. Despite this, the STAT3 inhibitor, WP1066, could be a viable candidate to address EV activation of latent HIV-1 as we showed it could selectively kill HIV-1-infected cells. Future experiments should focus on uncovering alternative mechanisms for EV activation of HIV-1 while also examining the feasibility of dasatinib and WP1066 as supplements to cART in vivo.

## Supplementary Materials

The following are available online at https://www.mdpi.com/1999-4915/12/6/665/s1, Figure S1: EV characterization using ZetaView, Figure S2: PBMCs lack caspase-3 and PARP-1, Figure S3: c-Src is present in extracellular vesicles (EVs) derived from different cell lines and EV populations
