# Supplementary material for: Extracellular Vesicle Activation of Latent HIV-1 Is Driven by EV-Associated c-Src and Cellular SRC-1 via the PI3K/AKT/mTOR Pathway
Source: Viruses. 2020 Jun 19;12(6):665. doi: 10.3390/v12060665 (PMC7354524; doi:10.3390/v12060665)
Supplement: Supplementary file 1 [file viruses-12-00665-s001.pdf]

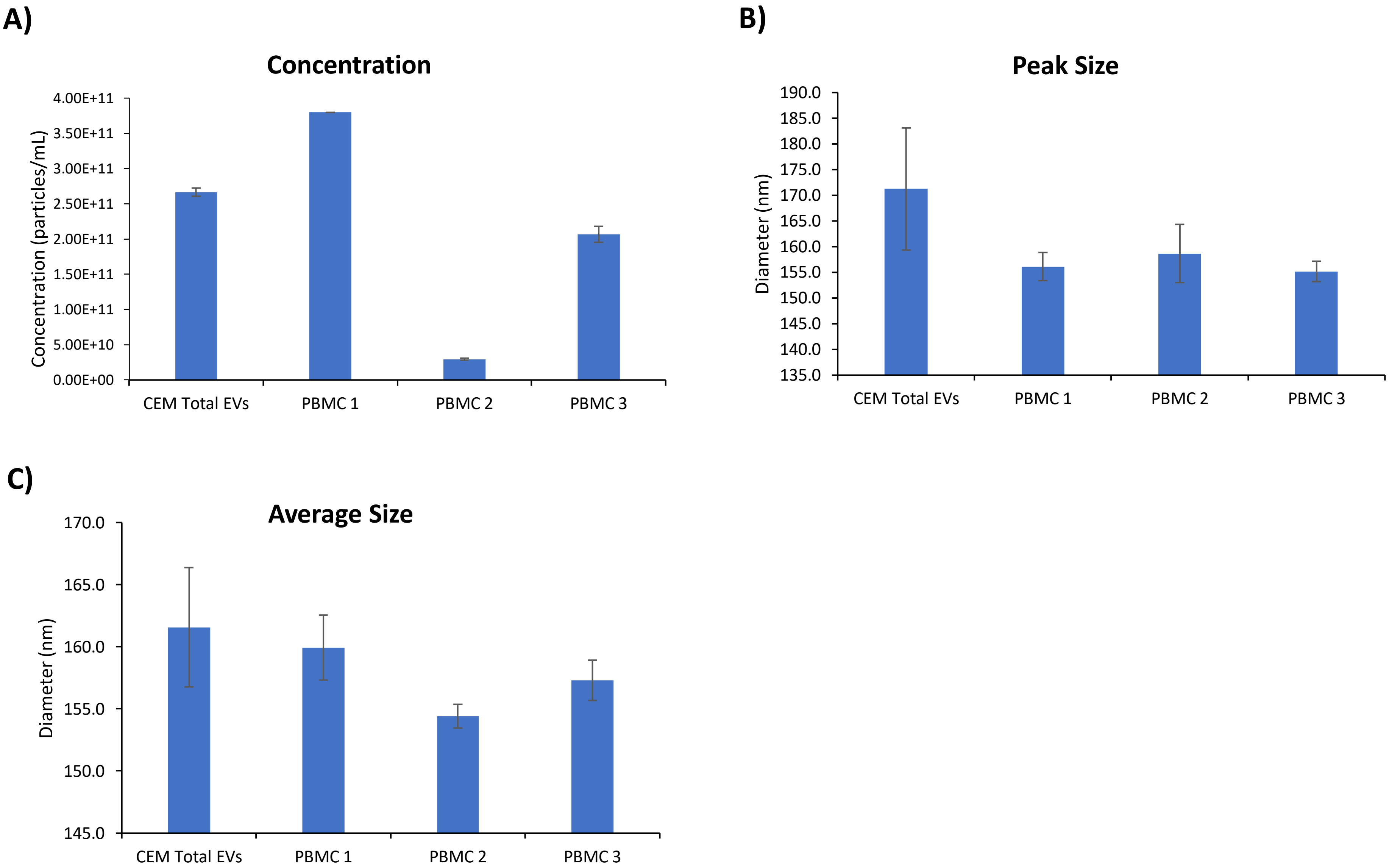

Figure S1: EV characterization using ZetaView. EVs were isolated from CEM cells and PBMCs via a 90-minute ultracentrifugation at 100,000 X g. Characterization of EVs including concentration (**A**), peak size (**B**), and average size (**C**) was performed using ZetaView NTA and analyzed with Microsoft Excel in three technical replicates for each sample.

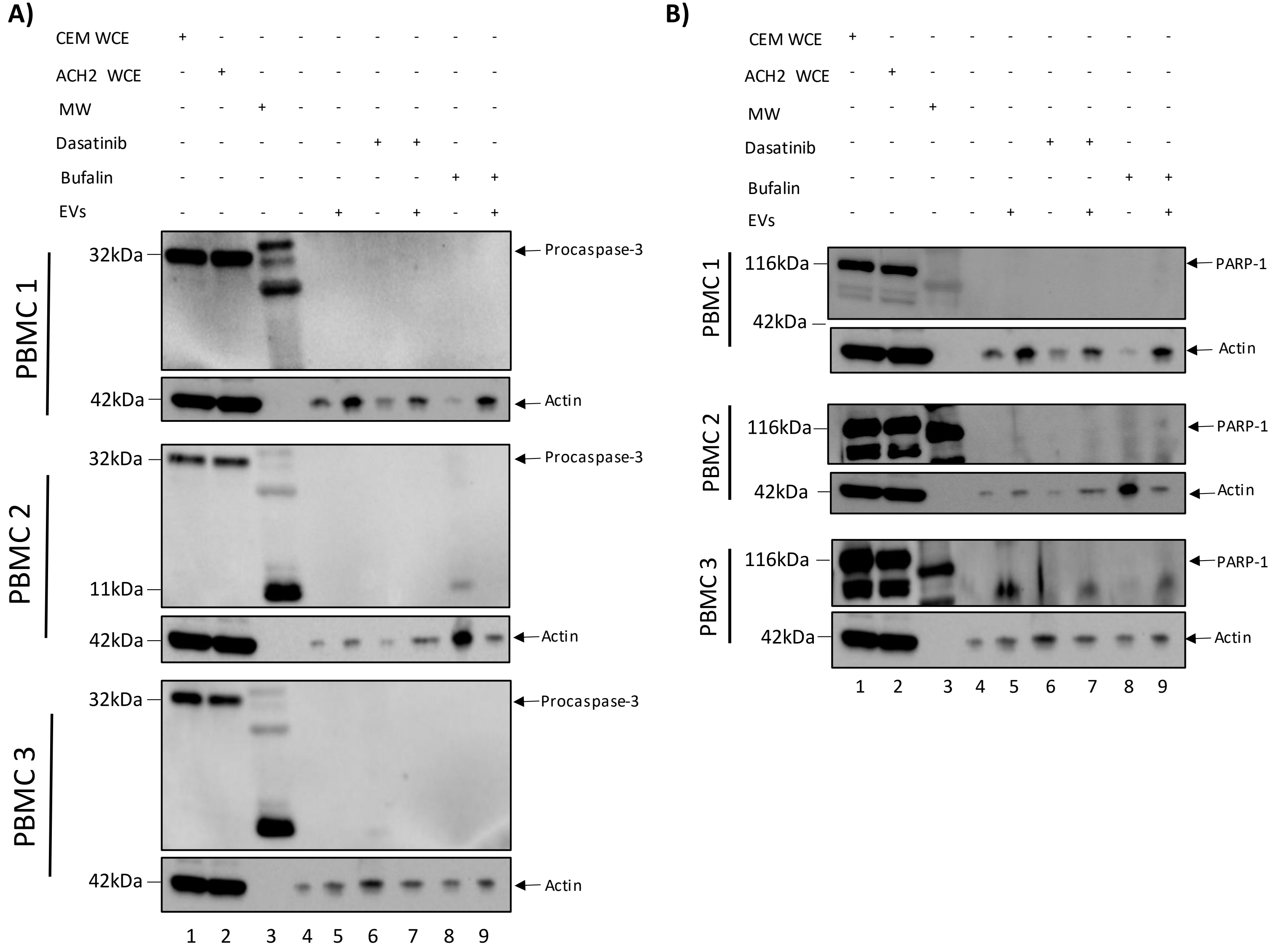

Figure S2: PBMCs lack caspase-3 and PARP-1. Three samples of HIV-1-infected, latent PBMCs were treated with 2.5 nM and 0.5 µM of bufalin and dasatinib, respectively, prior to incubation at 37°C for 2 hours. EVs isolated via ultracentrifugation from each of the PBMCs prior to infection with HIV-1 (89.6) were added back to each of their respective PBMCs. Cells were then harvested after 72 hours of incubation at 37°C. Virions were concentrated from cell supernatant using NT86 and NT 80/82 and rotated overnight at 4°C. Samples were Western blotted for caspase-3 (**A**) and PARP-1 (**B**). CEM and ACH2 WCE were used as a negative and positive control, respectively. Actin was used as a loading control.

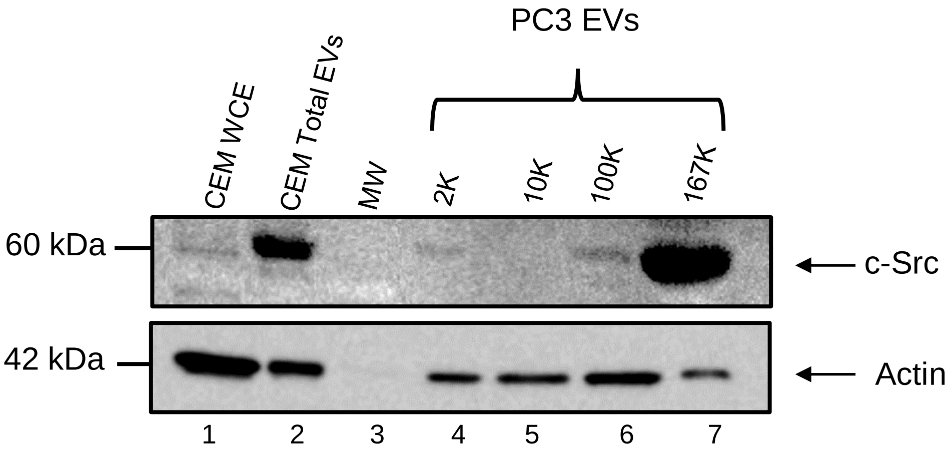

Figure S3: c-Src is present in extracellular vesicles (EVs) derived from different cell lines and EV populations. 2K, 10K, 100K, and 167K EV populations were isolated from PC3 cells via ultracentrifugation and Western blotted for c-Src. CEM whole cell extracts (WCEs) and CEM total EVs (comprising of 2K, 10K, and 100K EV populations) were used as positive controls, while actin served as a loading control.
